# Treatise on the Diseases of Women

**Published:** 1845-01

**Authors:** 


A PRACTICAL TREATISE ON THE DISEASES PECULIAR TO WOMEN. Illustrated by Cases derived from Hospital and Private Practice. By *Samuel Ashwell*, M.D., Member of the Royal College of Physicians, London; Obstetric Physician and Lecturer to Guy's Hospital. 8vo. Part III. London, 1844. Highley.

HAVING formerly spoken of this work very highly, our task will not now be a difficult one, especially as the present and concluding part is not only equal but superior in practical excellence and research to its predecessors. We have often had occasion, during the last twelve or fifteen years, to remark, that there was no lack of diligence or talent in the obstetric department of medicine, and the book of Dr. Ashwell, although we do not always agree with him, fully sustains its reputation. It is clear he intended it to be a safe guide in practice, and while he has proved himself thoroughly acquainted with the controverted pathology of female disease, he has avoided obscuring his own views by any forced attempt to make them harmonise with the opinions of others. His work is eminently his own; he has seen frequently that about which he has written; he has tested the worth of his diagnostic precepts by constant and accurate observation, and his treatment of the various maladies is evidently the result of most extensive and often very successful practice. Nor is there any dogmatism or vanity about his writing. The style is plain and forcible; without any dark passages, incomprehensible either by himself or his readers. In some places, however, Dr. Ashwell takes a little too much for granted; he supposes that others are as fully acquainted with female disease as he is, and therefore occasionally neglects a full exposition of his prognosis and remedial management.

We are glad Dr. Ashwell has had courage and candour enough to confess that, even with all the advantages of Guy's Hospital and a very extensive private practice, he has seen but few examples of some of the very rare diseases: this is as it should be, and affords a striking and instructive contrast to some of the French writers, who, as in M. Lisfranc's instance, exhaust the credulity of their readers by announcing the startling fact, or rather falsehood, that they have seen "hundreds of cases of a disease," (anteversion of the womb,) which, even in the largest fields of observation, occurs only extremely rarely.

We congratulate the author on another important point—the authentication of his cases. We are aware this cannot always be done, but certainly it is a matter of great moment. The case is the portraiture of the history of the disease, and as there are false facts in philosophy, so there may be false cases in medicine. Dr. Ashwell has given great additional authority to his work by an admirable selection of cases; the truth and reality of which no one can doubt. Let us not be misunderstood: we say again it cannot always be done; but we are sure it is often neglected where no real difficulty exists. Dr. Bright, a distinguished colleague of the author, and some other writers, have advantageously availed themselves of a similar verification of their reported cases, and we trust ere long to see it far more generally adopted.

The chapter on polypus contains an excellent summary, not only of the practice, but likewise of the pathology, of this important disease. Dr. Ashwell has not failed to embody some of the peculiar views of Lisfranc, nor has he hesitated to express his disapprobation of some of his measures. We agree with him in his incredulity about such frequent "enucleation," and in his condemnation of severe and violent efforts to abstract polypi while still high up in the uterine cavity. There is much force in Dr. Ashwell's remark, "such things don't require to be done in England." But we think the author is yet too cautious and careful about removal by the knife, as we are confident, in many instances, it is far better and safer than the ligature. We shall, as in our former reviews, allow the author to speak for himself in an extended analysis, assenting or dissenting as we proceed.

The following is his definition of polypus:—

"*A firm and insensible tumour, usually round and smooth, and growing by a*

*stalk either from the mucous lining of the uterus, or the structure beneath;* [illegible] *chief symptom being hemorrhage. It commences in the cavity of the womb, in t*[illegible] *channel of the cervix, or from the os. It is often of fibrous texture, not malignan*[illegible] *and rarely ulcerates. It is covered by mucous membrane, and sometimes by an a*[illegible]*ventitious coat, the product of inflammation. There is little pain, menstruation* [illegible] *excessive, and conception may occur."*

Dr. Ashwell observes that polypus of the uterus, while it is not [illegible] uncommon disease, is certainly far more rare than cancer. In the follo[illegible]ing remarks we entirely concur.

"There is no malady more certainly curable than polypus, although ma[illegible] patients have died from its accompanying bleedings, without its existence e[illegible] having been suspected, much less ascertained. The necessity for vaginal exam[illegible]nation, where profuse uterine bleedings resist the remedies employed for th[illegible] suppression, cannot be too strongly urged. In the hemorrhages of polyp[illegible] astringents are useless—the only effectual remedy is removal.

"Pain can scarcely be said to be a symptom, and the first suspicion of t[illegible] disease is excessive menstruation, or bleeding occurring in the catamenial inte[illegible]vals. Although the evacuation of the bladder or intestines is seldom prevente[illegible] it is not impossible that a large polypus, by pressure on the urethra or rectu[illegible] or both, may obstruct their functions. Hence, if the patient be strong, the lo[illegible] of blood seldom attracts notice, till some of its injurious effects begin to [illegible] realized. When the digestion becomes impaired and there is leucorrhœa, dea[illegible] sallowness, difficult respiration, and other evils,—then anxiety begins, and it [illegible] not long till a reluctant permission is granted to examine the state of [illegible] womb."

There is considerable variety in the size of these growths. Some, [illegible] larger than a garden bean, have bled alarmingly; while a polypus [illegible]ceeding a Seville orange in size, scarcely bled at all, ultimately produci[illegible] irritation by its pressure on the neck of the bladder, and great exhausti[illegible] by constant and large secretions of pus. We are glad Dr. Ashwell [illegible] dwelt on this fact, as the continuance of purulent discharge, and the alm[illegible] entire absence of hemorrhage, has induced most injurious delay in asc[illegible]taining, by a proper examination, the existence of the disease. The p[illegible]tracted inclusion of a polypus within the uterine cavity is perplexing [illegible] dangerous; as a small one, especially if it be soft and vascular, may [illegible] rise to alarming and even fatal losses of blood. We insert the follow[illegible] case entire, as it is an instructive one, and conveys a good idea of [illegible] author's clear and forcible style of writing.

"Such instances have occurred in my practice, and a hard polypus of mo[illegible]rate size, now in Guy's Museum, removed when it had very partially desce[illegible] through the cervix, fully attests the truth of the preceding observations. [illegible] polypus grew so slowly, probably because the hemorrhages had been freq[illegible] and excessive, that three years elapsed prior to its coming within the reac[illegible] the finger. During this period, the patient had been repeatedly seen by [illegible]nent obstetric physicians, who in vain attempted to restrain the bleedings. [illegible] first, as she believed it to be entirely useless, I was not permitted to make [illegible] examination; but on my assuring her, that although the growth had not [illegible] descended into the vagina, it might speedily do so, she consented; and I [illegible] then the opportunity of touching a small, hard, and insensible tumour, [illegible] emerging from the os. The bleeding which followed alarmed me so much, [illegible] before leaving the house, I attempted its removal. Excision was out of [illegible] question, and with a very long instrument, I made two unsuccessful efforts [illegible]fore I could apply the ligature. When completed, the canulæ were some[illegible] within the channel of the cervix. It was an anxious case, for there were sev[illegible]

bleedings within the first two days, and from one of them it did not seem for some hours that she would have rallied. During the twelve days the ligature was applied, the patient was never quite free from uterine pain. Often I had to loosen the whip-cord, to foment the abdomen with hot gin and laudanum, and twice a day to give an opiate. At length, however, to my great gratification, both the instrument and the polypus came away. The swollen legs and feet, the deadly paleness of the skin, and the universal anæmia gradually vanished, and the patient is now, after a lapse of several years, in confirmed health."

There can be no doubt, that women have died from hemorrhage, or the diseases resulting from loss of blood, where a polypus had really descended into the vagina, which might have been early and easily removed; and many have been lost from similar bleeding, where the growth was shut up in the uterine cavity, and *beyond* the reach of surgical assistance. M. Lisfranc would probably deny the accuracy of this last conclusion: Dr. Ashwell, we can easily suppose, would decline an operation in cases where Lisfranc would unhesitatingly attempt one. The former practitioner would dread inflammation and gangrene as the consequence of the necessary surgical violence, M. Lisfranc would proceed regardless of such a risk, trusting, for his justification, to the certainty of death from continued hæmorrhage if the polypus were not destroyed. We do not regard our author as an infallible guide on such a point, but we confess we had rather submit to his discretion, than resign ourselves to the headlong procedure of the French surgeon. Nevertheless we do think there are cases where fatal hæmorrhage might sometimes be averted by greater boldness and an earlier operation.

Dr. Ashwell has furnished a full and correct account of the various kinds of polypus, and the reader will obtain much valuable information from its perusal. The principal pathological conditions of these growths consist in their proneness to bleed, their insensibility, and their non-reproduction after removal. Our author regards the hæmorrhage from polypi as furnished by distinct blood-vessels existing in the growth itself, and of course communicating with those of the uterus. In the following remarks we concur.

"Polypi are devoid of nerves, and are therefore insensible. Occasionally, however, it is probable, that portions of the uterus grow into, and form a part of the morbid structure itself; thus accounting for the continued, and sometimes severe pain produced by the first application and subsequent tightening of the ligature. It is not difficult to imagine, where a polypus has originated in the structure of the uterus, deeper than the mucous membrane lining its cavity, that for a time it will be imbedded amongst the uterine fibres; but as it grows towards the cavity, these fibres being distended and thinned, will eventually give way; and the polypus will henceforth be covered almost entirely by mucous membrane, that portion of the polypus only nearest to the uterus being invested by the proper tissue of the organ."

"I may say, that I have very seldom tied a polypus where any bleeding occurred after the lapse of a few hours from the noosing. And further, that in only two or three instances after either tying or excision, has there been any alarming loss of blood.

"It is not easy to understand, if the bleeding did not arise from the polypus, how Dupuytren could be correct in asserting, that, after his many operations by excision, alarming hemorrhage scarcely ever occurred.

"Bleeding, after either ligature or excision, will probably depend on the condition of the uterine tissue surrounding the base of the polypus. If this and the structure beneath are healthy, there will rarely be hemorrhage; if, on the contrary, they are soft and highly vascular, bleeding may occur."

*Malignant Growths and Ulcerations of the Uterine Cavity.*—These are rare, if we except those arising during the progress of carcinoma; and the author remarks, that he has seen only two specimens of fungi in this situation unconnected with cancer. "In one, an out-patient at Guy's, which destroyed life by bleeding, the growth reached nearly to the cervix, being raised about a quarter of an inch above the surrounding tissue: but in most of these instances the productions were probably connected either with polypi, moles, hydatids, or cancer."

The treatment can only be palliative. "The strong alum hip-bath, (a pound of the salt to a gallon of water,) care being taken that the fluid passes up the vagina—is one of the best remedies. Many other auxiliary means may be tried; and if the diseased mass, or a large portion of it, shall pass into the vagina, it may be removed by ligature, or by any more suitable method."

*Spongoid Tumour, or Fungus Hæmatodes Uteri.*—Our author is not afraid to say he has seen but one example of this very rare disease. The *history*, he tells us, "differs from cancer, the enlargement not commencing in the cervix, but in the body, and rapidly, as in the case I had under my care, affecting the entire structure. If examined by the rectum, the uterus, much larger than natural, is felt to be lobulated and elastic, and without any induration. Its mischievous effects on the general health are early apparent: the pulse being quickened, the strength rapidly failing, and the stomach and other organs quickly giving way. The pain is said to be agonizingly severe, and occasionally lancinating: and, probably even before ulceration is set up, there are profuse sanguineous discharges: in their intervals there is an almost constant escape of an offensive, dark coloured, purulent fluid, which not unfrequently, by its acrimony, excoriates the pudendum. The pain increases as the disease advances, and the final termination is similar to carcinoma. In the case above alluded to, although there were many symptoms resembling cancer, the larger size of the uterus, its rising above the pubis, and the freedom of the vagina and rectum from induration, sufficiently establish the diagnosis. The os was capacious enough to admit one or two fingers; and ulceration had affected its posterior lip. As to *treatment*, it can only be palliative, and the observations on this subject, appended to the chapter on cancer, are equally applicable here."

*Ulceration of the Mucous Lining of the Uterus.*—This is the least common of the diseases to which the womb is liable; and Dr. Ashwell has not seen an example. These admissions without any attempt at concealment do the author great credit: indeed, in all the affections of the uterus, his opinion of their average occurrence may be safely taken. Guy's Hospital has been open to him for many years, and, together with an extensive practice, has afforded him excellent opportunities for a correct appreciation of their frequency. We are much pleased, too, with the manner in which Dr. Ashwell speaks of other writers. He is evidently desirous to procure for them a full and just share of praise, and we never find him withholding from their works the authority and weight they deserve. We shall follow his example by inserting, on account of its extreme rarity and value, the following narrative.

"Dr. Francis Ramsbotham preserved a preparation of this disease, in which the organ acquired the size of a pregnancy of the fourth month, and where, being turned inside out, it was seen to be everywhere ulcerated. The parietes were not more than a quarter their natural thickness, and there was a ragged aperture at the fundus, large enough to admit three fingers.

"Dr. Ramsbotham, senior, and Dr. Gooch seem to be the only authors who have noticed this affection, and it is somewhat singular, that they both record the same case, having seen it together. The following is the history of its progress, and of the appearances observed on dissection, as published by the son of the former distinguished physician:—

"The lady, the mother of a family, considered herself between three and four months advanced in pregnancy; but the abdomen was enlarged to a size equal to what it has usually acquired towards the close of gestation. When my father first saw her, the uterus was distinctly perceptible above the pelvis, large, firm, resistant, and acutely painful throughout its whole extent, on pressure being applied. One portion of it, within the right ilium, was more tender than the rest. She had a dejected countenance, and was suffering under fever, with great irritability of stomach, and excessive irritation over the whole surface of the skin. She had been the subject of a constant discharge from the vagina for the preceding five or six weeks, in greater or less quantity, sometimes perfectly sanguineous, at others more serous, but devoid of unpleasant odour. Her increase in size had been uniformly progressive, though rapid. As in her last pregnancy, a dropsical state of the ovum occurred, the inordinate enlargement of the uterus was now attributed to the same cause. She became worse, and Dr. Gooch saw her in consultation with my father and her other professional advisers. On an examination per vaginam being now made for the first time, doubts arose both in Dr. Gooch's and my father's mind, as to the correctness of her opinion that pregnancy had occurred. The cervix uteri was found elongated and thickened, the mouth soft, flaccid, and sufficiently open to admit the passage of the finger within it about half an inch, but no substance could be detected in the cavity. The treatment directed was merely palliative; and as the bad symptoms became aggravated, on another consultation, five days after the former, it was determined to introduce a catheter within the uterus, that the liquor amnii might be evacuated, provided it contained an ovum. The instrument passed high up without encountering any impediment or obstruction; it could be 'moved about, as if *in vacuo*.' A few hours after this means had been adopted periodical pains came on, with a little increase of uterine discharge: these ceased spontaneously, in a short time exhaustion supervened, and the same day she died.

"On inspecting the body after death, it was remarked that the abdomen was tumid, and soft under the hand, having lost its former firmness. The peritoneal cavity contained a quantity of offensive gas, which escaped on the parietes being divided. The uterus was as large as though six months of pregnancy had elapsed. Its external surface was preternaturally red; it was flabby in texture, and, on squeezing it, some blood escaped through the vagina, mixed with puriform and serous fluid. The parietes were softened, and had much of the appearance of the gravid state. The cavity, which would easily have held the head of a child at birth, contained no fœtus, nor any other substance that could be looked upon as the result of impregnation. The whole internal membrane was destroyed by ulceration, and the surface was granulated. Adherent to the back part of the body was found a shreddy fibrinous mass, the size of a large egg, entangled among the irregularities of which were coagula and a quantity of bloody puriform matter. At different points near the cervix, the structure was eaten through nearly to the peritoneal covering.

"With regard to the treatment of such a case, we know so little of its nature, that I can only recommend you to palliate whatever dangerous symptoms may arise. If, indeed, we were quite sure the disease under our care was of this kind, astringent fluids injected into the uterine cavity by a properly contrived syringe, might induce a more healthy action, and, perhaps, in the early stage, be productive of essential benefit."

*Physometra of the Uterus.*—The author has never seen a true case of tympanites—one where the air has been the product of morbid secretion from the uterine vessels, and where, from the closure of the os, it has been allowed to collect for weeks or months in the uterine cavity, and has then, either spontaneously or by operation, been expelled; but he has several times been called on to cure explosions of gas from the vagina, which, forming in the uterus, escaped involuntarily, and with so much noise as to prevent the sufferer from venturing into society. This, we apprehend, is the usual extent of the disease, notwithstanding the many *singular* examples mentioned by different authors.

"It is said, that air has been known to accumulate in the uterine cavity after the death of the fœtus, or between the amnion and chorion, the fœtus being alive; and Baudelocque was present, *where the gaseous exhalation occurring after death, was sufficient to expel the child!!*" The author may well express his surprise and incredulity; such things do not occur in this country, although it is in some respects a land of wonders.

Peter Frank, a name of high repute, relates an example, where, after death, the uterus was hard, enlarged, and elastic, and full of gas of a very fætid smell. There was also ulceration in the cavity, and the neck was indurated. In another case, the os was closed by a polypoid growth. By the same author it is stated that, in the wife of a German physician, the accumulation of gas was so great, that the womb reached from the pubis to the diaphragm.

In a patient of Dr. Gooch's, pregnancy always cured the disease; while, in another, the uterine origin of the gas was confirmed, by the fact, that the instant pregnancy occurred the malady ceased, returning a few weeks after delivery.

"Idiopathic uterine tympanites is no doubt an exceedingly rare disease. Physometra, on the contrary, dependent on chemical change in the secretions, although a rare, is a more common affection. Thus the menstrual fluid, the vaginal and uterine mucus, coagula resulting from menorrhagia or dysmenorrhœa, the ichor of cancer, portions of placenta or of polypi, may, by their partial or entire decomposition, give rise to larger or smaller quantities of gas. A few months ago I had to remove a large mass of partially adherent placenta, which for three weeks subsequent to labour had caused frequent and large hemorrhages. On entering the uterine cavity, which was partially blocked up by a firm coagulum, Mr. Woolnough, a student of Guy's Hospital, and myself, were surprised by the escape of an immense quantity of fætid gas, doubtless the consequence of the putrefaction of the retained viscus."

"*Treatment.*—In cases where such accidental circumstances have not led to the cure of the disease, or where the gaseous accumulation causes severe and extensive pain, nausea, and vomiting, or difficult breathing, the introduction of a canula, or a long and elastic, yet firm male catheter, will certainly open a channel for its escape. How long the instrument should remain will depend upon the evacuation of the air, and on the likelihood of irritation and inflammation; nor will the management be quite so simple, if adherent masses of placenta, polypoid or fungoid growths, are the causes of the disease. Some authors, in order to effect a permanent cure, advise the injection of the cavity of the womb with warm water, weak solutions of chlorine, and chalybeate and astringent lotions. My present experience, independently of the frequent dangerous results of such uterine injections, would lead me to believe that they can very seldom be necessary."

*Hydrometra, or Dropsy of the Uterus.*—This, like physometra, is a rare disease, but, unlike the tympanitic affection, is often dangerous. Dr. Ashwell makes some interesting observations on aqueous discharge after parturition: indeed the whole of this section deserves careful study. During the last ten years, neither amongst the in nor the numerous outpatients of Guy's Hospital has there been a single case; a tolerably satisfactory proof of the extreme infrequency of the affection. The early *symptoms* comprise indigestion, nausea, and vomiting, flatulence, pain, and costiveness. Early in the *idiopathic* variety, the accumulated fluid is generally serous or mucous, containing albumen, thick and inodorous; but as the disease advances the contained fluid becomes dark, thick and offensive. In the *symptomatic* hydrometra, the dropsical secretion must of course frequently be mixed with blood or pus. "Authors," says Dr. A., "differ as to the quantity of fluid, the more reasonable assigning the moderate measures of pints and quarts, as the usual extent of the dropsy; while abroad, *where wonders are more common,* (and in this remark we concur) Blanchard, in one case, found eighty-five pounds of an ichorous and oily fluid; Vesalius 180 lbs.; and Bonet, who need not fear competition in the marvellous, relates an instance, *where the uterus, under this disease, was capable of holding a child six years old!*" "Credat Judæus."

"*Pathology.*—From what has been said as to the *varieties* of hydrometra, it will be inferred that the pathological conditions associated with it must be different. Thus, in Dr. A. T. Thompson's case, the uterus was perfectly healthy, with the exception of a sphacelated portion of the peritoneal covering of the fundus: while in Mr. Coley's, of Bridgnorth, the womb was entirely diseased: both these interesting narratives I shall annex, not only because they are authenticated by two well-known practitioners, but because they furnish the best description of the commencement, progress, and termination of the malady which I have yet read.

"However complicated, therefore, may be the diseased states of the womb, two conditions are essential to hydrometra: first, that there should be increased secretion from the lining membrane, or from some growth or ulceration of its surface; and, second, that there should be impermeability of the channel of the cervix.

"Burns says, that one large hydatid filling the cavity of the organ, constitutes the malady; and Denman once saw an empty cyst of the form and size of the uterus, expelled after the discharge of the dropsical fluid it had contained. It is clear that the former celebrated writer has not assigned the true cause.

"*Treatment.*—For this it is sufficient to refer to physometra, the evacuation of the fluid, the prevention of future accumulation, and the re-establishment of the health being the points of especial consequence."

*Of Uterine Moles.*—This term is not accurately defined. All fleshy and shapeless masses, irregularly passing from the uterus, have been thus designated; but the author says, they may originate from the ovum, which has been early blighted, or which has been only imperfectly developed; from a portion of retained placenta; from the firm clots of dysmenorrhœa; from a polypus spontaneously detached and shut up in the uterine cavity; from fibrous portions of coagulated blood, or from the hardened mucus of the uterus itself. There are, therefore, two kinds of uterine moles, viz. those which are the product of conception; and those which are independent of pregnancy. Of course the majority of such cases may be traced to conception as their first cause; but it is also certain that there are moles and hydatids which do not thus originate. Moles differ much from each other; sometimes not resembling any animal form, but rounded with an external coating like skin. The author remarks that there are several examples in Guy's Hospital Museum of the mole which has been termed "the false germ," where the embryo is absent, while the membranes are somewhat perfectly formed.

"All pathologists allow the existence of those moles, however differently they may explain the circumstance of their formation, where the embryo having died early, the ovum being retained, has increased in size and solidity, not by a process of growth, as in natural pregnancy, nor even as in a tumour or polypus, but by the effusion of coagulable lymph from inflammation of the lining membrane. This forms successive layers over the surface of the dead ovum, giving it eventually a great degree of consolidation. Some of these masses, when cut into, have no cavity; but the chorion and amnion are demonstrable, although the enveloping lymph may be one or two inches in thickness. It seems somewhat surprising, that the coverings of the fœtus, should be carefully constructed when there is no embryo. But the fact is so. Lately, I was present at the expulsion, after much previous flooding, of a firm, fleshy mass, equalling in size a large orange. The small central cavity was lined by a smooth and perfectly formed amnion, with a little fluid; but, although I examined the specimen under water most carefully, I could detect no appearance either of an embryo or umbilical cord. If in such instances the embryo has never been formed, they may be regarded as genuine examples of false conception. Some physiologists however, have supposed that, in these cases, the tender germ may have been accidentally and early deprived of life, and subsequently dissolved in the liquor amnii. However explained, the absence of the embryo is thus certified."

*Of Moles which do not owe their existence to Conception.*—The author has twice seen fibrous clots, the product of dysmenorrhœa, growing into mole, not expelled till they had attained a considerable size, and then only with great pain and serious hæmorrhage. The following case we give in Dr. Ashwell's own words.

"Some years ago, I was asked by Dr. Hodgkin to visit a lady a few miles from town, who was thought to have polypus. On examination, a fleshy and tolerably firm body could be touched first within the cavity of the cervix uteri, there had been considerable bleeding, and the anemia was distressing. Ergot was given, and in a few days the mass was protruded through the os. A ligature was placed around it, which in twelve hours cut through, bringing away the tumour, but not without considerable hemorrhage. Ergot was again exhibited, forty minims of the tincture every quarter of an hour, and after the sixth dose, a fibrous mass, as large as a turkey's egg, of firmly coagulated, and partially organized blood, was expelled. In six or seven weeks another mass, only smaller, was got rid of in the same way. This lady had long suffered from dysmenorrhœa, and had frequently passed firm concrete clots of lymph and blood. There had been no sexual intercourse for eighteen months prior to this occurrence. She afterwards died; dropsy of the chest and abdomen having supervened."

*Vesicular Moles or Hydatids of the Uterus.*—We are tempted to enquire why these products are not classed amongst the moles that are dependent on pregnancy. We can scarcely believe, even on Dr. Ashwell's high authority, that these vesicles ever originate merely in diseased action of the lining membrane of the uterus: certainly the chorion to which they are attached, and from which as a root they grow, can only exist as the product of conception. The chorion, unlike the decidua, is an entirely fœtal production, and we are therefore constrained to believe, that these vesicular hydatids, invariably found in connection with it, must be fœtal, also; or rather the blighted remains of the embryo. But Dr. Ashwell shall speak for himself.

"*Pathology.*—These formations are placed in the second species of moles, because I have seen at least one example where they were the result of diseased action of the uterine lining membrane, independently of sexual intercourse. The patient was the widow of a surgeon, and of undoubted reputation. Her husband had been dead two years and a half when the abdomen began to enlarge. She had nausea, but no vomiting, from which she had always suffered in her pregnancies. The increase of size was very rapid, and at three months and a half from the first stoppage of menstruation, the bulk of the uterus had reached that of a seventh month's pregnancy. The abdominal tumor was flaccid, and the os closed. At the fourth month, after more than ordinary exertion, there was a gush of blood from the vagina, followed by the immediate escape of a considerable quantity of vesicular hydatids.

"The recovery was good. Iron was afterwards given, she was sent to the sea-side, and now, at the expiration of several years, there has been no return of the malady.

"Mr. Douglas Fox, surgeon to the Derbyshire Infirmary, gave me the particulars of a case where a large mass of vesicular hydatids was expelled from the uterus of a maiden lady, where the hymen was unruptured, and of whose chastity there could not be a suspicion.

"Sir Charles Clarke and Dr. Blundell unite in opinion, that conception is not a necessary condition; while Madame Boivin, Capuron, Duges, and even our own countrymen, Denman and Burns, have arrived at an opposite conclusion. Dr. Evory Kennedy says, that 'hydatids may occur in virgins'; while Dr. Montgomery believes, 'that they invariably result from impregnation.' It were to be wished that every disputed physiological point admitted, as this does, of a settlement by the observation of facts.

"Women are liable to a repetition of this vesicular formation, where it has resulted from conception. The few exceptions, where the hydatids have formed independently of pregnancy, forbid at present any decided opinion as to the probability of their recurrence."

We regret, that want of space compels us to pass over several chapters of great practical value, especially the long and most able section on the various displacements of the uterus: in future editions we shall remedy this omission. At present we must content ourselves by a long extract, in every word of which we agree, on the now fashionable and frequently entirely unwarrantable operation of ovariotomy. We are truly glad, that Dr. Ashwell has given to the profession this most temperate and accurate opinion, and has thus thrown the weight of his great experience and reputation into the scale against this most formidable and unsuccessful operation. We prognosticate that, like extirpation of the uterus, in a few years extirpation of the ovary will be scarcely heard of, except as a matter of medical history.

"The progress and termination of encysted ovarian dropsy, have become subjects of the deepest interest, owing to the efforts lately made to cure the disease by extirpation. Whether this be a desirable, or even a defensible operation, must mainly depend on the known course of the disease, when either left to itself or treated with a view to palliation only. If it could be proved, in the majority of cases, that the malady did not shorten life nor induce severe suffering, few more operations would be undertaken. But the examples of this kind are, it is to be feared, only exceptional; and yet I cannot divest myself of the idea, if our records were accurately kept, that a more favourable view might be correctly taken of the palliative, or indeed of any treatment which did not involve the necessity for this hazardous extirpation. Certain it is, that *many* women have lived to old age, who were the subjects of the disease; and although a less number comparatively survive many years after tapping has become necessary, yet a collection even of these would go far to prove, that paracentesis is not by any means so fatal in this respect as has been supposed. Sabatier examined the bodies of several women who had carried these encysted tumors during half a century, without alarming derangement of health; and the memoirs of the French Academy of Surgeons prove, that it may last 58 years; while Nauche, as a summary of his own views, says, 'dropsy of the ovary, then, is not a very alarming disease, unless it be very ancient and very voluminous.' The cases of frequent tapping recorded by Martineau, Portal, and many other surgeons, amply attest the protracted duration of life in association even with this stage of the affection. Nor, in a calculation of this kind, must it be forgotten, that numerous women have become pregnant, and have been many times safely delivered, notwithstanding a dropsy of one of the ovaries. Such cases have fallen under my own observation, and I could add others also where the malady, although of a considerable size, has existed many years without tapping, and without indeed any other than mere palliative treatment.

"These considerations are entitled to great weight when determining the propriety of extirpation, uncalled for by present and great evils; or where the operation, from the enthusiastic views of its patrons, is urgently recommended as a preventive of mischiefs which they deem, but not always on good grounds, to be prospectively inevitable. To operate, where the patient strongly desires it, from a conviction that her sufferings and the frequent repetitions of paracentesis, will otherwise prove speedily fatal, may not involve any distressing responsibility, especially where the condition of the tumor leads to the supposition, that the case is pathologically a favourable one. But there are examples selected for operation far different from this. Take, for instance, a case which occurred to me a few months ago. A lady travelled to town from a considerable distance, anxious to have extirpation performed. On inquiry, I found she was 62 years of age, had never been tapped, although ovarian dropsy had existed for more than half her life. There was scarcely any suffering beyond weight and pressure, although the tumor was of immense size and partly solid. In such a case, it would have been highly culpable to have operated; and yet a surgeon, over zealous about the removal of ovaries, had induced the firm belief that it ought to be done. I need scarcely add that the patient, after being made acquainted with the great danger of the operation, was perfectly satisfied to remain as she was. Nor will the practitioner be less perplexed and distressed by such a case as the following, which occurred within my observation not long since:—A young woman, under 22, had ovarian dropsy; her countenance bespeaking excellent health, and her history confirming the impression. Without interference, many years might have been added to her existence; and as one of the fortunate incidents of life, it might have so happened, that the tumor should cease to grow. But unhappily she was convinced that extirpation was proper; the operation was most ably performed, and in a few days she died. These certainly are not the cases in which removal ought to be practised. If the operation is to become established, of which I have the strongest doubt, it must be confined to examples of the malady where tapping has already been so often performed as to preclude, from the experience of similar cases, any idea that it can ever be dispensed with; and where, we are confident, that great suffering must lead to early death. Perhaps this may be regarded as too limited a view of the value of extirpation, but it is, I think, the correct one. In such cases, if the diagnosis excludes the belief that there are serious adhesions, or malignant and solid growths complicating the tumor, and if the patient strongly desires it, the operation is defensible. In all other examples it can only rest on the patient's own views of her future prospects, and on a calculation of chances. She might live many years and without much suffering; but she may die in a few years after great suffering: she determines, therefore, being courageous, and probably strongly urged by her surgeon, to run the risk of immediate death for the hope of immediate and radical cure. Whether she has done wisely to submit to such a hazard, a successful operation can scarcely prove; that she has happily secured her safety, through imminent peril, such an operation does prove. Lithotomy, operations for hernia, and for securing large arteries, rest on different grounds. That they are essential to the patient's life, is a full justification of their performance; for in all, even if not dangerous at the moment, it is certainly known that life will soon be destroyed, either by fever, gangrene, or loss of blood. Such, it has been proved, has not been the case in many of the fatal operations lately performed for extirpation of ovarian encysted tumors. It does not appear that statistics more favourable even than we have any right to expect, will materially change the aspect of the circumstances under which this operation is to be performed. It must, probably, from the impossibility of determining the real character and adhesions of the growth, ever remain an eminently uncertain operation. The extirpation, we are assured, by the operators themselves, in a fit case, is far from difficult—would that it were more so —for then it would not be so readily undertaken. If it required as much surgical knowledge and skill to make these large and brilliant abdominal incisions, as to tie the subclavian artery or to perform a trying operation of lithotomy, the lives of many women would have been already spared, and fewer would be sacrificed for the future. What would be thought of the feasibility of any other operation involving life in the most imminent hazard, if we discovered that out of 67 cases where it had been attempted, it was, *from absolute error of diagnosis, incapable of completion in eighteen;* that of the remaining 49 patients, where the extirpation was effected, *sixteen died* and *two were not cured;* so that out of the whole number 67, *the operation failed in thirty-six and succeeded in thirty-one,* less than one-half. Such results are distressing, especially when we hear no greater doubt expressed about the operation itself, but only higher confidence in its value, and greater laudation of the operators. We willingly concede presence of mind and ability to many of the extirpators of ovarian cysts; but we are unable to discover (for the later operations have been quite as unsuccessful from unfitness of the cases as the earlier ones) that any advance has been made in diagnosis. Nor, when the tumors themselves are examined after death, when the malignancy of many of them is recognized, and their firm, almost indivisible adhesions, and their immoveable masses of new and morbid substance are brought to view; it is next to impossible to entertain any sanguine hope, that our means of diagnosis can ever be much improved."

In concluding our still imperfect analysis, we must, in justice to the author, declare our conviction, that his work on female diseases is the most able, and certainly the most standard and practical, we have yet seen. It will, now that it is completed, find its way into the library of every practitioner, and justly confer on its talented author a very high place in the first class of obstetric physicians.

Nor can we close these critical remarks, without congratulating Dr. Ashwell, that even his unfinished book should have received from our utilitarian and clear-sighted brethren in America the high compliment, not merely of republication for sale, but of an extensive reprint for private circulation, amongst the members of the largest Medical Society in the United States: Dr. Hall, the Secretary, remarking, " that it may afford Dr. Ashwell some gratification to know, that his labors are appreciated on this side of the Atlantic, and are conferring a special benefit on six or seven hundred physicians in Massachussetts."

